# Diagnostic Performance of SARS-CoV-2 Rapid Antigen Test in a Large, German Cohort

**DOI:** 10.3390/children8080682

**Published:** 2021-08-08

**Authors:** Olivier Mboma, Elmar Rieke, Parviz Ahmad-Nejad, Stefan Wirth, Malik Aydin

**Affiliations:** 1 Center for Child and Adolescent Medicine, Center for Clinical and Translational Research (CCTR), Helios University Hospital Wuppertal, Witten/Herdecke University, 42283 Wuppertal, Germany; olivier.mboma@helios-gesundheit.de (O.M.); stefan.wirth@uni-wh.de (S.W.); 2 Institute for Medical Laboratory Diagnostics, Center for Clinical and Translational Research (CCTR), Helios University Hospital Wuppertal, Witten/Herdecke University, 42283 Wuppertal, Germany; elmar.rieke@helios-gesundheit.de (E.R.); parviz.ahmad-nejad@helios-gesundheit.de (P.A.-N.); 3 Laboratory of Experimental Pediatric Pneumology and Allergology, Center for Biomedical Education and Research, School of Life Sciences (ZBAF), Faculty of Health, Witten/Herdecke University, 58448 Witten, Germany

**Keywords:** COVID-19, SARS-CoV-2 early diagnosis, rapid antigen detection test, primary healthcare center

## Abstract

We assessed the performance of a rapid antigen test (RAT) in everyday clinical practice. Between 1 November 2020 until 1 April 2021 all in-patients at the Helios University Hospital Wuppertal, Germany, as well as the accompanying relatives at the Children’s Hospital received a SARS-CoV-2 RAT and a SARS-CoV-2 RT-PCR prior to admission. Out of 3686 patients, 22 (0.6%) subjects were tested positive by RT-PCR and RAT, and 3591 (97.4%) were negative by both methods, showing discordant results: RT-PCR+/RAT− in 58 (1.6%) and RT-PCR−/RAT+ in 15 patients (0.4%). Overall sensitivity and specificity of RAT was 27.5% (95%CI 18.1–38.6%) and 99.6% (95%CI 99.3–99.8%), respectively. The sensitivity was slightly higher in adults (30.4%, 95%CI 18.8–90.9%) than in pediatric subjects (20.8%, 95%CI 7.1–42.2%). False negative RAT had a statistically higher Ct-value (*p* < 0.001) compared to true positive values, and overall sensitivity increased to 80% [59.3–93.2%] with Ct value < 30. While the sensitivity of the RAT was poor compared with the RT-PCR, the specificity was excellent. However, the sensitivity increased with lower Ct value, and with the right anamnesis the RAT can be a quick and easy approach to distinguish people who are infectious with SARS-CoV-2 from noninfectious people, enabling appropriate triage in clinical practice while waiting for the RT-PCR result.

## 1. Introduction

Emerging infectious diseases that spread as pandemics do not only influence the morbidity and mortality of affected populations but also have a major socioeconomic and political impact [1,2]. Since the beginning of the 21st century, three separate outbreaks of coronaviruses (CoVs) have caused global public health crises: the Severe Acute Respiratory Syndrome (SARS) in 2002–2003, the Middle East Respiratory Syndrome (MERS) in 2012, and SARS-CoV-2/COVID-19 (Coronavirus Disease 2019) since winter 2019 [3,4,5]. The clinical presentation of the infection may vary from barely noticeable flu-like symptoms to severe pneumonia with acute respiratory distress and death [4].

SARS-CoV-2 is a single-stranded RNA virus with a virion diameter of 60–140 nm [5]. On the basis of its phylogenetic cluster formation, it belongs to the genera of the β-coronavirus [6]. Approximately 50% of its genome sequence is identical to MERS-CoV, and an 80% match exists with SARS-CoV [7]. Referring to the numbers from the Johns Hopkins University (Baltimore, MD, USA, July 2021), this virus is associated with over 4.1 million deaths worldwide [8]. In Germany, over 3.7 million people have been infected with the virus, with over 91,000 deaths related to the virus being recorded [9].

Therefore, a rapid and reliable diagnostic method is crucial in combating the epidemic, as it enables infected individuals to be identified quickly and correctly. This allows for timely isolation and adequate therapy, which contributes to significant containment of the pandemic. It was shown during the SARS-CoV pandemic in 2003 that control measures delayed by one week not only resulted in the outbreak lasting four weeks longer, but that it also increased quantitatively by almost three times [10].

During this pandemic, adults have been the main affected group with higher morbidity and mortality than children [11,12,13]. Furthermore, it appears that children play a minor role in the dynamics of the pandemic and, therefore, fewer data are available on performance of the SARS-CoV-2 antigen rapid test in the pediatric population [13,14,15].

At our facility, all admitted patients received both reverse transcription polymerase chain reaction (RT-PCR) and a rapid antigen test (RAT) regardless of clinical symptoms or signs of COVID-19. The antigen test can be performed with little effort and provides the result after approx. 15 min, whereas the PCR results take up to 24 h, depending on the capacity of the laboratory [16,17].

This enables an advanced triage in clinical practice and prevents further infection, as all patients with a positive antigen test are isolated until the (RT-PCR) test result is available.

This project was designed to determine the diagnostic test accuracy of the SARS-CoV-2 antigen rapid test in everyday clinical practice and to compare the performance in adult and pediatric subjects.

## 2. Materials and Methods

### 2.1. Study Cohort and Recruiting Period

We performed a single center, observational study, which was conducted between 1 November 2020 until 1 April 2021 at the Helios University Hospital Wuppertal, Witten/Herdecke University, Germany. All in-patients, as well as the accompanying relatives at the Children’s Hospital received a SARS-CoV-2 RAT and a SARS-CoV-2 RT-PCR prior to admission. On the day of the test, a form was filled out in advance by the guardians. The form consisted of item parameters regarding clinical symptoms associated with SARS-CoV-2, as well as possible contacts who had been tested positive for the virus. Relatives who had a temperature ≥ 38.5 °C or were under quarantine at the time of admission were not included and had to be replaced by another adult family member.

The study was approved by the Ethics Committee of Witten/Herdecke University, Germany (S-09/2021).

Each patient admitted to the hospital received a total of two sequential swabs. For each swab, the throat was swabbed first and then the nasopharynx. (MANTACC™, Miraclean Technology Co., Ltd., Shenzhen, China). One of the swabs was used for the rapid antigen test, while the other was placed in 3 mL of universal transport medium (Glucose-Lactalbumin-Yeast Virus (GLY), Xebios Diagnostics GmbH, Düsseldorf, Germany) and sent to our laboratory to carry out the RT-PCR testing.

### 2.2. Diagnostic Procedures

For the RAT, we used the VivaDiag™ SARS-CoV-2 Ag Rapid Test Device (VivaCheck Biotech (Hanghzou) Co., Ltd., Hangzhou, China) and the Panbio™ COVID-19 Ag Rapid Test Device (Abbott Rapid Diagnostic Jena GmbH, Jena, Germany). Both tests were qualitative membrane-based immunoassay based on the Colloidal Gold Immunochromography principle and target the nucleocapsid protein of SARS-CoV-2 in nasopharyngeal samples.

For the isolation of RNA, we used Maelstrom (Taiwan Advanced Nanotech Inc. (TANBead), Taoyuan, Taiwan) or NIMBUS (Seegene, Seoul, Korea). The RT PCR was performed using different Seegene assays following the manufacturer’s instructions (Allplex™ 2019-nCoV Assay, Allplex™ SARS-CoV-2 Master Assay, Allplex™ SARS-CoV-2/Flu A/Flu B/RSV Assay), or assays on the NeuMoDx 288 (Qiagen N.V., Hilden, Germany).

### 2.3. Clinical Data

Demographic (age and sex) and clinical variables of the study population were obtained from electronic medical records retrospectively.

### 2.4. Statistical Analysis

Continuous variables were given as median with interquartile range (IQR), and the categorical variables as proportions. Differences between groups were compared using the Mann-Whitney U-test or Fisher’s exact test. Receiver operating characteristic (ROC) curves and Youden’s index were used to determine the SARS-CoV-2 RT-PCR cycle threshold (Ct) cut-off value best discriminating between RT-PCR+/RAT+ and RAT− subjects. A two-sided *p* value of 0.05 or below was considered significant for these comparisons. Statistical analysis was performed with SPSS v26.0 (IBM Corp., Armonk, NY, USA).

To assess the diagnostic test accuracy of the RAT, the SARS-CoV-2 RT-PCR was established as the gold standard. Sensitivity (Se), specificity (Sp), positive (PPV) and negative predictive value (NPV) were calculated with a 95% confidence interval (CI). Cohen-Kappa statistics were used to calculate the agreement between the various test procedures.

## 3. Results

### 3.1. Patient Characteristics

A total of 3686 patients were included during this period, 876 (23.9%) of whom were children and 2810 (76.2%) were adults. Of these, 61.3% were female. The median age distribution of the children was 6 years (interquartile range (IQR) 1–14 years) and 43 years (IQR 31–71 years) for the adults. The largest group in this population were adults between 18–34 years old. Clinical characteristics were collected solely in case of positive RT-PCR SARS-CoV-2 result (Table 1 and Table 2).

### 3.2. Performance of the Rapid Antigen Test

Out of 3686 patients, 22 (0.6%) subjects tested positive and 3591 (97.4%) tested negative through RT-PCR and RAT. Interestingly, both methods showed discordant results: RT-PCR+/RAT− in 58 patients (1.6%) and RT-PCR−/RAT+ in 15 patients (0.4%) (Appendix A Table A1). The overall concordance between the two methods was fair (κ 0.37, 95% CI 0.26–0.48). However, this was moderate (κ 0.42, 95% CI 0.28–0.56) for adults and fair for pediatric patients (κ 0.26, 95% CI 0.07–0.45). Overall Se and Sp of RAT was 27.5% (95%CI 18.1–38.6%) and 99.6% (95%CI 99.3–99.8%), respectively. The sensitivity was slightly higher in adults (30.4%, 95%CI 18.8–90.9%) than in pediatric subjects (20.8%, 95%CI 7.1–42.2%) (Table 3). The Se was also higher in symptomatic subjects (52.9%) compared to asymptomatic subjects (20.6%) (Appendix A Table A2). Se in the different pediatric age groups was highest in 5 to 14 years age group at 25%, followed by 23.1% in 0 to 4 years age group, and 14.3% in 15 to 17 years age group (Appendix A Table A3). With a prevalence of 2.2%, overall RAT positive and negative predictive values were 59.5% (95%CI 44.2–73.1%) and 98.4% (95%CI 98.2–98.6%), respectively. The positive predictive value was higher in adults (70.8%, 95%CI 51.2–84.9%) than in pediatric subjects (38.5%, 95%CI 18.1–63.9%) (Table 3).

The range of cycle threshold (Ct) values of the nucleocapsid (N)-gene were 12.2–39.9 (median 31.9 IQR 24.3–36.8), 14.3–38.5 (median 29.5 IQR 22.6–34.5) for the envelope (E)-gene and 15.9–39.9 (median 30.8 IQR 23.0–36.8) for the RNA-dependent RNA polymerase (RdRP)-gene.

**Figure 1:**
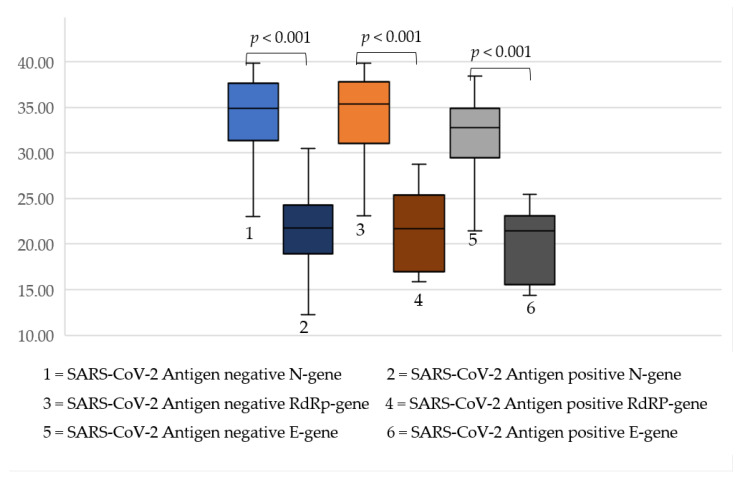
Distribution of the different Ct values from people who tested either RAT+ or RAT−.

**Figure A1:**
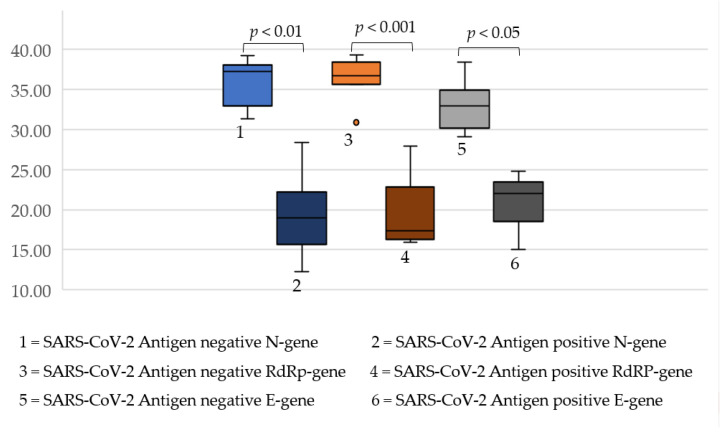
Distribution of different Ct values from people tested either RAT+ or RAT− in the pediatric group.

**Figure A2:**
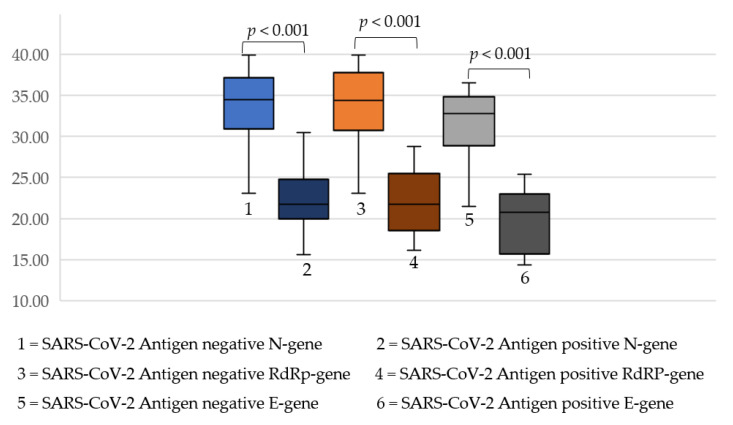
Distribution of different ct values from people tested either RAT+ or RAT− in the adult group.

There were statistically significant differences when comparing the Ct values between patients with a false-negative and those with a true-positive RAT (Figure 1). This remained the same in the pediatric population (*p* < 0.05) and in the adult population (*p* < 0.001) (Appendix A Figure A1 and Figure A2).

**Figure 2:**
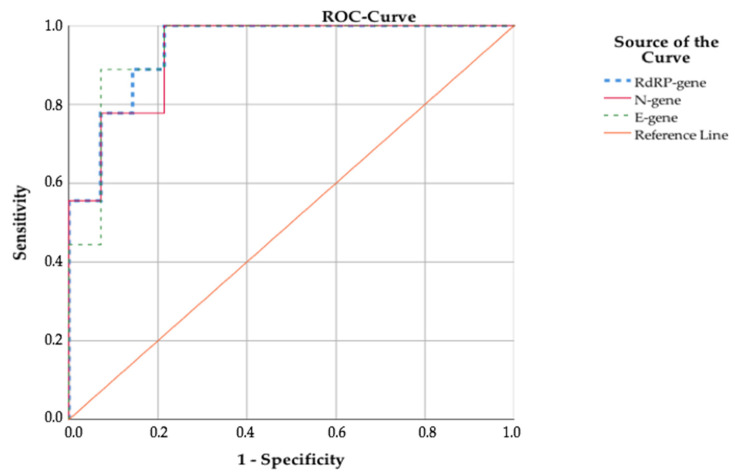
ROC-curves of the different Ct values.

In our study, ROC curves and Youden’s index analyses indicated that for the N-, E- and RdRP-gene, the optimum cut-off of the different Ct values which discriminated best between RT-PCR+/RAT+ and RT-PCR+/RAT− subjects, were <30, <25 and <30 respectively. The sensitivities were 100% (N- and RdRP-gene) and 88.9% (E-gene). The specificities were 78.6%, 92.9% and 78.6% (Figure 2). 

**Figure A3:**
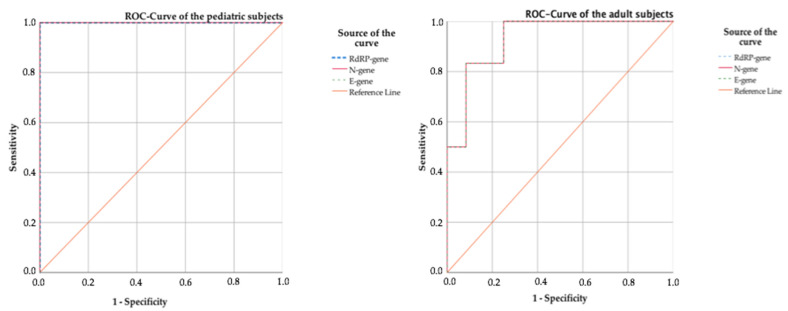
ROC curves of the different Ct-values for the pediatric and adult subjects.

In the pediatric group, the Ct cut-off values were <30 (N- and RdRP-gene) and <27 (E-gene) with overall Se and Sp of 100%. For the adult subjects the Ct cut-off values stayed the same with a sensitivity of 100% and specificity of 75% (Appendix A Figure A3). As expected, the total RAT sensitivity was directly dependent on the Ct values (Appendix A Table A4).

To quantify the performance of the RAT in the context of community-based testing, a calculated cut-off for the N-gene Ct value of < 30 was used. This is also the threshold for high virus concentrations that corresponds to the range considered most transmissible. The overall population Se increased to 80% (95%CI 59.3–93.2%) with 75 % (95%CI 50.1–91.3%) for the adult group and 100% (95%CI 47.8–100%) for the pediatric group (Table 4).

## 4. Discussion

The primary goal of controlling the COVID-19 pandemic is to reduce the transmission of infection in the population by reducing the basic reproduction number (R0). The R0 is determined by different factors, such as the duration of infectiousness or the number of susceptible individuals in the population [18]. Diagnostic technologies play a vital role in infectious disease control as they help to identify and isolate infected individuals and thus prevent further spreading of infection [19].

As of 10 May 2021, the European Commission department of Health and Food Safety has presented a common list of 83 SARS-CoV-2 RAT of which 35 the Member States have agreed to mutually recognize their test results for public health measures [20]. Over 400 RAT Kits have been listed by the German Federal Institute for Drugs and Medical Devices [21].

A characteristic of our study population is that accompanying relatives in the children’s hospital who had a temperature ≥ 38.5 °C or had any symptoms related to COVID-19 at the time of admission were excluded. All other subjects from the other medical units were tested, even if they had symptoms of COVID-19 or not. That allowed the calculation of sensitivity and specificity values with greater relevance for daily clinical practice. In our study group, there were more women in the adult group (64.8%), as most of the children were admitted with their mothers.

The sensitivity and specificity of the SARS-CoV-RAT were published in a technical data sheet of the test to be 95% and 100% for VivaDiag™ SARS-CoV-2 Ag and 98.1% and 99.8% for Panbio™ COVID-19 Ag, respectively [22,23]. In this study, the two test kits were not separated from each other because it was no longer possible to determine which kit was used for which subject.

The overall specificity in this study was very high at 99.6% (99.8% adults vs. 99.1% pediatrics), and similar to that of the manufacturer’s information. This is consistent with previous studies describing the excellent specificities of different RATs ranging from 87.1–100% [24,25,26,27,28].

However, the sensitivities measured by the manufacturers differed markedly from the value determined by us. Here, the overall sensitivity was 27.5% (30.4% adults vs. 20.8% pediatric). One explanation for this could be that the manufacturer used a large number of samples with a high viral load in the analysis, not reflecting the distribution of the general population.

In clinical practice, overall SARS-CoV-2 RAT sensitivity has been reported to vary between 30.2% and 97% depending on the kit used [26,28,29,30]. These studies were performed in a population with different SARS-CoV-2 prevalence, ranging from 5% to 40% with large fractions of asymptomatic subjects [25,26,31,32]. In addition, the lower sensitivity of the RAT in this study could be partly explained by the high proportion of asymptomatic persons in the study population. The sensitivity was higher in symptomatic persons, with 52.9%, than in asymptomatic with 20.6%. These results are also confirmed by other studies, which suggest that the Se in symptomatic people can be twice as high as in asymptomatic people [33,34,35,36]. The prevalence in this study was 2.2%, possibly because this study was conducted during a major lockdown in Germany, and also because relatives with positive symptoms of a SARS-CoV-2 infection were not allowed to be admitted. Depending on the period and region, prevalence has been described to vary from 0.9% up to 80% [25,26,28,37].

Our results showed a greater sensitivity in adults (30.4%) than in pediatric subjects (20.8%). While there was no age-related difference in SARS-CoV-2 RNA load in the upper respiratory tract in other studies, one retrospective study in the Netherlands showed that viral load increases significantly with age [26,38,39]. However, direct comparison between studies is difficult due to differences in test kits used, clinical characteristics, age of patients, and types of samples processed.

In this study 15 (0.4%) of the RAT results were false positive. In retrospect, it was found that five of these 15 showed up on the same day and that the manufacturer’s instructions were not carried out correctly. Instead of three drops of the extraction solution, more drops were applied. Another source of error for false positive rapid antigen test is explained by the fact that weak bands were often subjectively assessed as positive in order to avoid incorrectly assessing patients as RAT negative and to prevent further spread of the infection. One study found that optimal performance was achieved with significant reductions in false positive rapid antigen tests when the bands were rated positive when spanning the entire width of the strip, regardless of the intensity of the band [30]. A training tool has been developed for this purpose [40].

The overall NPV of the SARS-CoV-2 RAT in this study was excellent, at 98.4%, and there was no significant difference in the pediatric or adult subjects, and in cases with low or high Ct-values. Similar high NPVs were also described in previous studies [26,41,42]. However, the clinical interpretation of NPVs should be made with caution, since predictive values depend on the prevalence of the disease and can therefore be deceptive. The negative predictive values are high especially in regions with low prevalence [43].

The Ct-dependent evaluation of all three genes shows that false negative rapid antigen test had a statically higher Ct value (Figure 1). In this study, using the ROC curves and Youden’s index analyses, the optimum cut-off of the different Ct values for the N-gene, E-gene and RdRP-gene which discriminated best between RT-PCR+/RAT+ and RT-PCR+/RAT− subjects were <30, <25 and <30, respectively.

The overall increasing of sensitivity to 80% (adults 75% vs. pediatric 100%) for positive SARS-CoV-2 RAT with a Ct value < 30, and thus with higher viral load, has been described in previous studies, with reports going up to 98% [24,25,26,27,32,44,45].

However, the cultivation of SARS-CoV-2 does not seem to be possible at a threshold of 10^6^ copies/mL [46]. Depending on the RAT-Kit used, and the laboratory, this represents a different Ct value cut-off. In some studies, this has been described as being >29–31, so that an infection with a high Ct value in the late stage of the infection could have little effect from a public health perspective [47]. A quantitative reference sample was established by the Robert Koch Institute, Department for Infectious Diseases and Center for Biological Hazards and Special Pathogens/Highly Pathogenic Viruses, and the consulting laboratory for coronaviruses at the Institute for Virology of the Charité–Berlin University of Medicine in Germany [48]. Using this, our laboratory determined the Ct cut-off value for the E-, RdRP- and N-gene, that corresponds to a viral load of 10^6^ copies/mL were 21.6, 24.5 and 25.2, respectively [49].

Nevertheless, in our study, 3% of the potentially infectious people (Ct values < 25.2) received negative RAT results. This means that a small number of infectious people were not recognized. In a cohort in Norway with over 4500 subjects tested, this number was 15% [25]. It is also important to note that the viral load of a sample does not necessarily reflect the viral load in the subject’s respiratory tract. It is also not sufficient to assess a patient’s contagiousness with the Ct value only, because this is influenced by other factors, such as the time since the onset of symptoms.

To put this in the context of child public health, serial testing is critical to compensate for the lower sensitivity of RAT, as asymptomatic children with high Ct levels at an early stage of infection may not be detected until later in the course.

The strengths of this study lie in the relatively large number of participants, and that it demonstrates the accuracy of a RAT in the context of everyday clinical practice.

Nevertheless, the results of the study should be considered in light of its limitations. First, we excluded all relatives who had symptoms associated with SARS-CoV-2 infection, which also explains the low prevalence in our study. The incorrect compliance with instructions and misinterpretation of test results can be interpreted as possible limitations of our study. In addition, we used Ct values as a surrogate for viral load and didn’t determine whether asymptomatic patients with high Ct values were in the early or late stage of infection.

To avoid underestimating the viral load of the patient, only efficient sample collection methods can deliver meaningful results. For that, adequate swabs must be made available, especially in pediatrics, which are fine enough to adapt to the child’s anatomy.

## 5. Conclusions

In summary, we conclude that although the overall sensitivity of the RAT is inferior, the specificity is excellent compared with the RT-PCR in adult and pediatric populations. With the right clinical history, the RAT can be a quick and easy approach, while waiting for the RT-PCR result, to distinguish people who are infectious with SARS-CoV-2 from noninfectious people, and thus enable appropriate triage in clinical practice and reduce further spreading of the virus in the clinical environment. Nevertheless, it is important to note that the excellent NPV of RAT, especially at times of low prevalence, is misleading, and that tools are needed with the highest possible sensitivity in the field.

## Figures and Tables

**Table 1:** Demographic and clinical distribution of patients.

	Adults ≥ 18 Years (*n* = 2810)	Pediatric < 18 Years (*n* = 876)
Demographic characteristics		
Gender (female)	1820 (64.8%)	440 (50.2%)
Age in years, Median [IQR]	43 (31–71)	6 (1–14)
Clinical characteristics of positive RT-PCR SARS-CoV-2 tested (*n* = 80 *)		
Fever > 38.5 °C	7 (14.3%) *n* = 49	4 (16.7%) *n* = 24
Cough	6 (12.2%) *n* = 49	3 (12.5%) *n* = 24
Rhinitis	6 (12.2%) *n* = 49	4 (16.7%) *n* = 24
Anosmia and/or ageusia	0 (0%) *n* = 49	1 (8.3%) *n* = 12
Diarrhea	0 (0%) *n* = 49	2 (8.3%) *n* = 24
Vomiting	0 (0%) *n* = 49	0 (0%) *n* = 24

* Some clinical information of the person who tested positive for RT-PCR was missing because not all forms were correctly filled out and the persons could not be contacted retrospectively.

**Table 2:** Age distribution of the patients (*n* = 3686).

Age in Years	Number of Subjects	Gender (Female)
0–4	410 (11.1%)	185 (45.1%)
5–14	283 (7.7%)	151 (53.3%)
15–17	183 (5.0%)	104 (84.1%)
18–34	1010 (27.4%)	913 (90.4%)
35–59	751 (20.4%)	460 (61.2%)
60–79	639 (17.3%)	247 (38.7%)
>80	410 (11.1%)	200 (48.7%)

**Table 3:** Performance of the Rapid antigen test in field.

	Overall Study Population	Adults ≥ 18 Years	Pediatric < 18 Years
Se	27.5% [18.1–38.6%]	30.4% [18.8–44.1%]	20.8% [7.1–42.2%]
Sp	99.6% [99.3–99.8%]	99.8% [99.5–99.9%]	99.1% [98.2–99.6%]
PPV *	59.5% [44.2–73.1%]	70.8% [51.2–84.9%]	38.5% [18.1–63.9%]
NPV *	98.4% [98.2–98.6%]	98.6% [98.3–98.8%]	97.8% [97.3–98.2%]

* Positive predictive value (PPV) and negative predictive value (NPV) were calculated using a prevalence of 2.2%.

**Table 4:** Performance of the rapid antigen test depending on the N-gene Ct value.

	TOTAL Ct Value < 30	TOTAL Ct Value ≥ 30	≥18 Years Ct Value < 30	≥18 Years Ct Value ≥ 30	<18 Years Ct Value < 30	<18 Years Ct Value ≥ 30
Se	80% [59.3–93.2%]	2.2% [0.1–11.8%]	75% [50.1–91.3%]	3.23% [0.1–16.7%]	100% [47.8–100%]	0% [0–23.2%]
Sp	99.6% [99.3–99.8%]	99.6% [99.3–99.8%]	99.8% [99.5–99.9%]	99.8% [99.5–99.9%]	99,6% [98.2–99.6%]	99.1% [98.2–99.6%]
PPV	57.1% [43.7–69.6%]	6.3% [0.9–33.1%]	68.2% [49.5–82.4%]	12.5% [1.8–53.0%]	38.5% [23.9–55.5%]	0
NPV	99.9% [99.7–99.9%]	98.8% [98.7–98.8%]	99.8% [99.6–99.9%]	98.2% [98.9–99.0%]	100.0%	98.4% [98.3–98.4%]

## Data Availability

The data to this study can be shared upon reasonable request from the first and corresponding authors.

## Appendix A

**Table A1:** Agreement between rapid antigen test and RT-PCR.

	SARS-CoV-2 RT-PCR+	SARS-CoV-2 RT-PCR−	
SARS-CoV-2 rapid antigen test+	22 (0.6%)	15 (0.4%)	37 (1.0%)
SARS-CoV-2 rapid antigen test−	58 (1.6%)	3591 (97.4%)	3649 (99.0%)
	80 (2.2%)	3606 (97.2%)	3686 (100.0%)

**Table A2:** Overall performance of the RAT for asymptomatic and symptomatic subjects.

	Overall Asymptomatic Subjects	Overall Symptomatic Subjects
Se	20.6% [11.5–32.7%]	52.9% [27.8–77.0%]
Sp	99.6% [99.3–99.8%]	99.6% [99.3–99.7%]
PPV	46.4% [30.1–63.6%]	37.5% [23.4–54.1%]
NPV	98.6% [98.5–98.8%]	99.8% [99.6–99.9%]

**Table A3:** Sensitivity and specificity of the RAT in different pediatric age groups.

	Pediatric 0–4 Years	Pediatric 5–14 Years	Pediatric 15–17 Years
Se (95% CI)	23.1% 5.0–53.8%	25.0% 0.6–80.6%	14.3% 0.4–57.9%
Sp (95% CI)	98.7% 97.1–99.6%	99.3% 97.4–99.9%	99.4% 96.9–99.9%

**Table A4:** Overall sensitivity of the RAT according to the different Ct-values.

**N-Gene Ct-Value**				
	<23	≤25	≤30	≤35
Se (95% CI)	100% 76.8–100%	88.9% 65.3–98.6%	76.9% 56.4–91%	45.7% 30.9–61%
**RdRP-gene Ct-value**				
	<23	≤25	≤30	≤35
Se (95% CI)	100% 71.5–100%	91.7% 61.5–99.8%	76.2% 52.8–91.8%	53.5% 34.4–71.7%
**E-gene Ct-value**				
	<21	≤25	≤30	≤35
Se (95% CI)	100% 54.1–100%	84.6% 54.6–98.1%	60% 36.1–80.9%	38.7% 21.9–57.8%
